# Stress Fracture of the Femoral Neck Following Total Knee Arthroplasty: A Case Series

**DOI:** 10.7759/cureus.36702

**Published:** 2023-03-26

**Authors:** Anil Arora, Getnet Asnake, Venktesh Sonkawade

**Affiliations:** 1 Orthopaedics and Joint Replacement, Max Super Speciality Hospital, Delhi, IND

**Keywords:** stress fracture of femur neck, total knee arthroplasty, osteoporosis, bone mineral density, rheumatoid arthritis

## Abstract

A stress fracture of the femoral neck after total knee arthroplasty (TKA) is rare, with few reported cases in the English literature. We defined a stress fracture following TKA as a nontraumatic fracture developing in the femoral neck within six months of TKA. This retrospective case series highlights the predisposing factors, diagnostic challenges, and management of stress femoral neck fractures following TKA. In our series, the major risk factors for the fracture are an increase in the level of activity in osteoporotic bone after a period of relative immobility after TKA, steroid intake, and rheumatoid arthritis. Preoperative dual-energy X-ray absorptiometry (DEXA) screening may help in the early initiation of osteoporosis treatment as the majority of our cases come late in the knee arthritis stage and long after a period of relative inactivity. Timely diagnosis and management of a stress femur neck fracture in the early period may prevent fracture displacement, avascular necrosis, and nonunion.

## Introduction

A stress fracture occurs in a normal bone because of repeated occurrences of mechanical loading far below the bone's ultimate strength [1]. These fractures may be the final stage of fatigue or insufficiency of the affected bone [1].

The occurrence of a stress fracture of the femoral neck following total knee arthroplasty (TKA) is rare [2-5]. Many authors have described it as being with old age, female gender, less preoperative mobility, an increased level of activity on the osteoporotic bone after TKA, a high body mass index, rheumatoid arthritis, prolonged steroid intake, severe deformities of the knee treated with a rotating hinge prosthesis, and a change in the mechanical axis of the hip and knee after correction of severe deformities in TKA [2-6]. The early diagnosis of a stress fracture might be challenging because of the insidious onset of symptoms and normal findings on an early radiograph, requiring advanced imaging modalities to confirm the diagnosis [7]. Management of stress fractures of the femoral neck depends on the MRI morphology of the fracture and findings of intraarticular hip effusion. Incomplete compression-sided fractures with less than 50 percent of the fracture line and no intraarticular hip effusion are treated conservatively with six weeks of nonweight bearing followed by a gradual return to full weight bearing as tolerated. Incomplete compression-sided fractures with hip effusion, incomplete compression-sided fractures with more than 50 percent fracture line, tension-sided fractures, and displaced fractures are treated operatively [7]. Unrecognized stress fractures of the femur might result in displacement of the fracture, nonunion, avascular necrosis, and undesired joint replacement surgery [7,8].

We report five cases of stress fractures of the femur neck that developed within six months after TKA. A single surgeon operated on all the TKAs through the median parapatellar approach using cemented unconstrained (cruciate retaining or posteriorly stabilized) knee implants. The main aim of this paper is to focus on the early diagnosis and various management strategies at different stages of stress fractures of the femoral neck following TKA.

## Case presentation

Case 1

**Figure 1:**
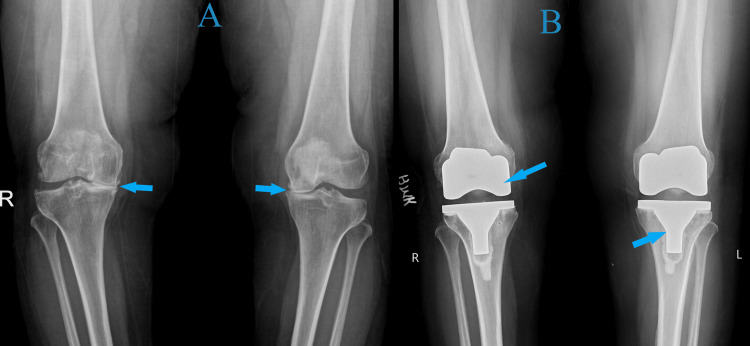
A) Preoperative X-ray showing varus deformity and arthritic bilateral knee joints. B) Immediate postoperative X-ray-aligned and well-positioned total knee arthroplasty implants.

**Figure 2:**
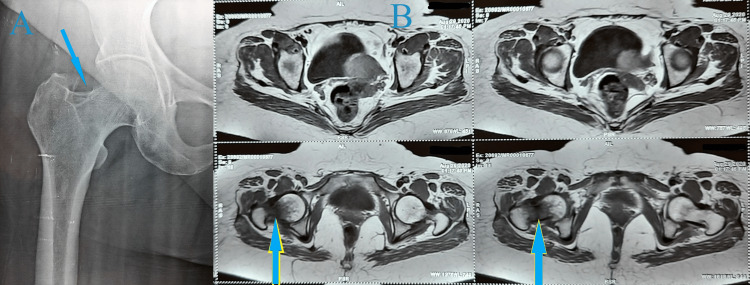
A) Right hip X-ray showing a nondisplaced stress femur neck fracture on the tension side of the cortex. B) MRI showing bone edema and stress fracture of the right femur neck

**Figure 3:**
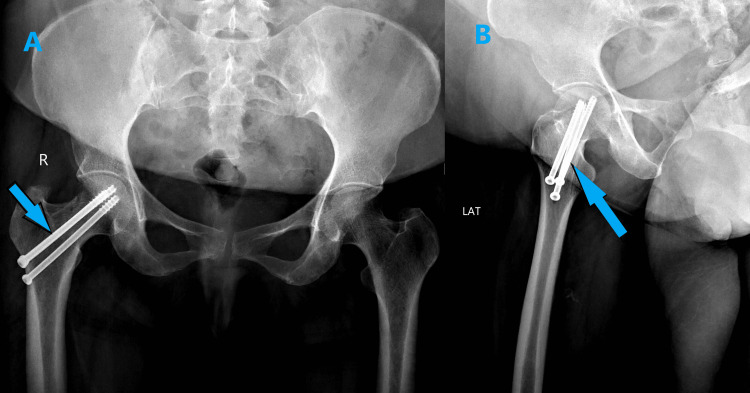
Immediate postoperative radiograph showing right femur neck stress fracture fixed with cannulated hip screw (A&B)

**Figure 4:**
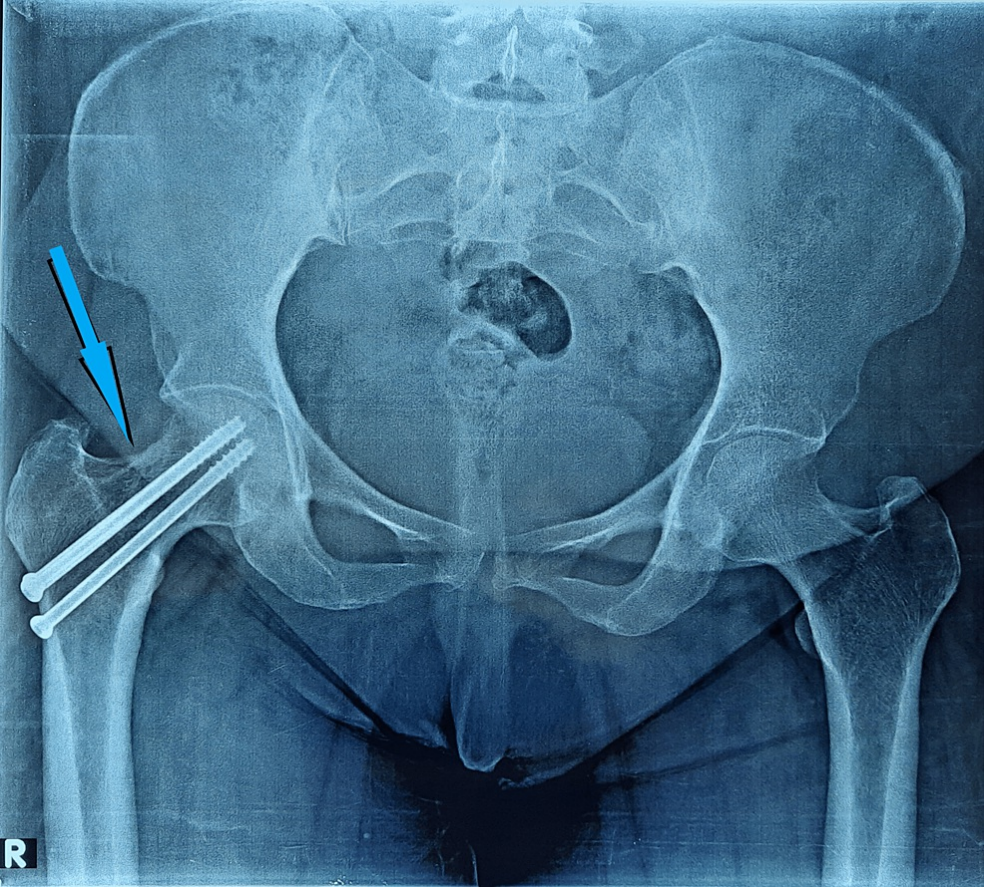
A six-month follow-up radiography showing a healed fracture of the right femur neck stress fracture

A 55-year-old female, 78kg in weight, 158cm in height, and 31.2 kg/m2 BMI with rheumatoid arthritis in both knees, underwent bilateral TKA using a cemented cruciate-retaining prosthesis (Depuy Synthes, PFC Sigma Implants, Warsaw, IN) (Figures 1A-1B). She was taking a low dose of steroids for a prolonged period. After surgery, the rehabilitation course was uneventful, and she could engage in light activities without significant pain. Despite denying a history of trauma, she experienced an insidious onset of right hip pain during weight-bearing four months after TKA. A radiograph of the hip showed a sclerotic line along the tension side of the cortex of the right femur neck, suspicious of a stress fracture (Figure 2A). An MRI revealed a right femoral neck stress fracture (Figure 2B). DEXA of the hip and lumbar spine showed a T-score of -2.6 and -1.8. We managed operatively with cannulated hip screws to prevent displacement (Figures 3A-3B). Six months after surgery, she was pain-free and could return to normal activities, and an X-ray revealed a healed fracture (Figure 4).

Case 2 

**Figure 5:**
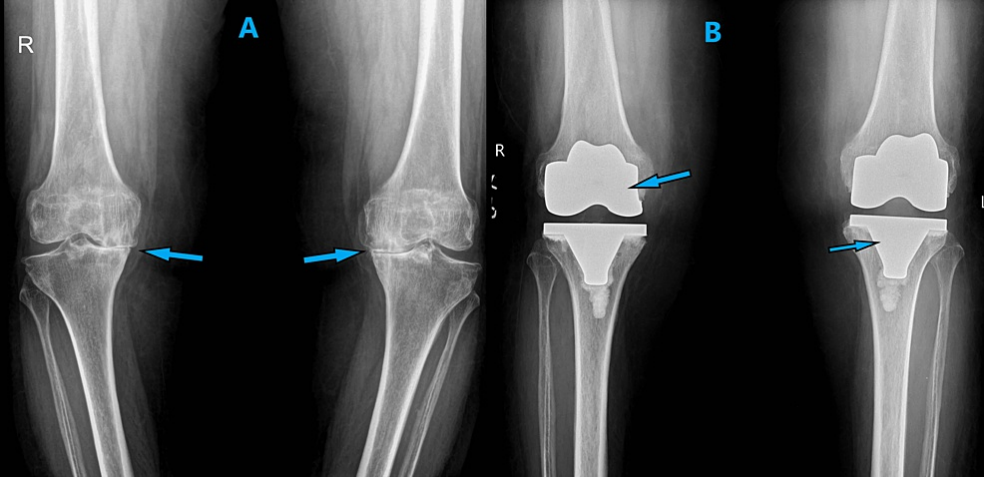
A) Preoperative X-ray of both knee joints showing features of arthritis and varus deformity. B) Postoperative X-ray showing good alignment and positioning of total knee arthroplasty implants

**Figure 6:**
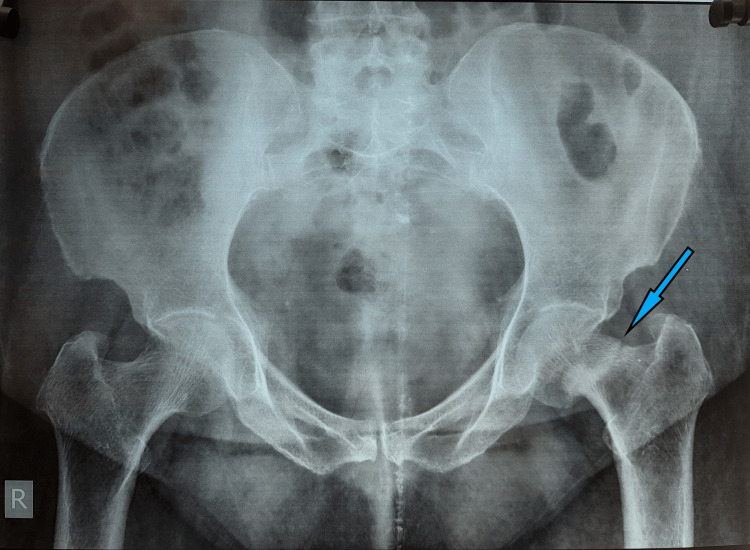
Pelvic X-ray showing a sclerotic line running along the left femur neck, suggestive of a stress femur neck fracture

**Figure 7:**
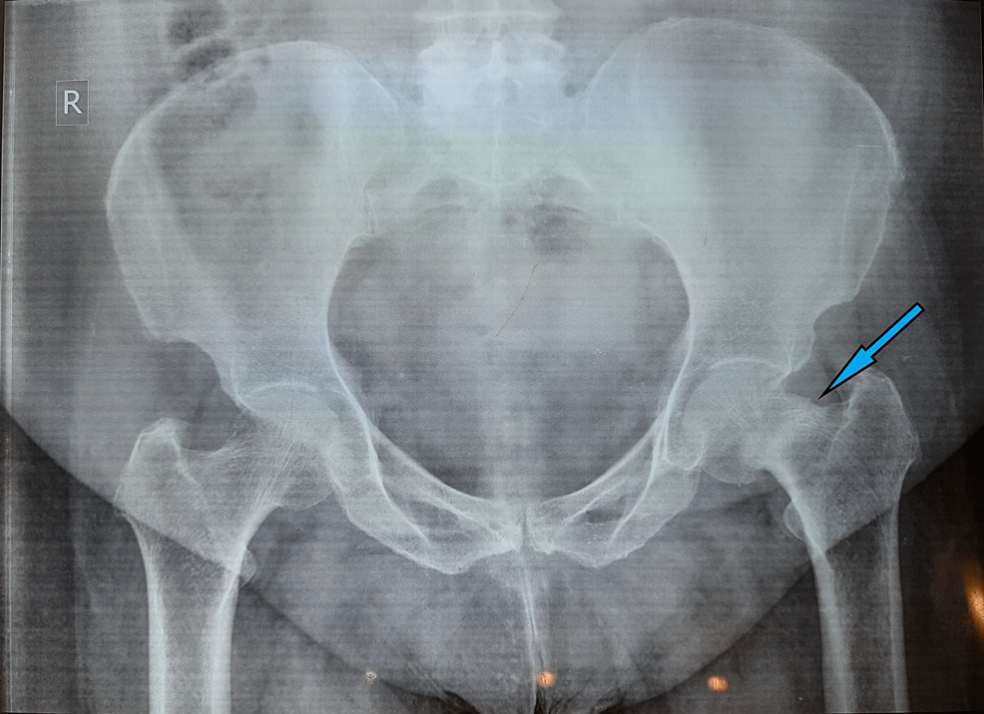
A six-month follow-up pelvic X-ray, showing healed fracture of the left femur neck stress fracture after nonoperative treatment

A 60-year-old female, 83kg in weight, 155cm in height, and 34.5kg/m2 BMI with bilateral knee osteoarthritis underwent TKA using a cemented cruciate-retaining prosthesis (Opulent, Meril, India) (Figures 5A-5B). Three months after TKA, she had no pain and was ambulating comfortably. After four months following TKA, she had increased left groin pain and a limp while walking. An X-ray of the hip showed a non-displaced stress fracture of the left femur neck (Figure 6). DEXA of the hip and lumbar spine showed a T-score of -2.7 and -2.0. She refused surgery but agreed to complete bed rest for two months, followed by gradual weight-bearing. Six months after the fracture, an X-ray showed signs of fracture healing, and she resumed normal activities (Figure 7).

Case 3

**Figure 8:**
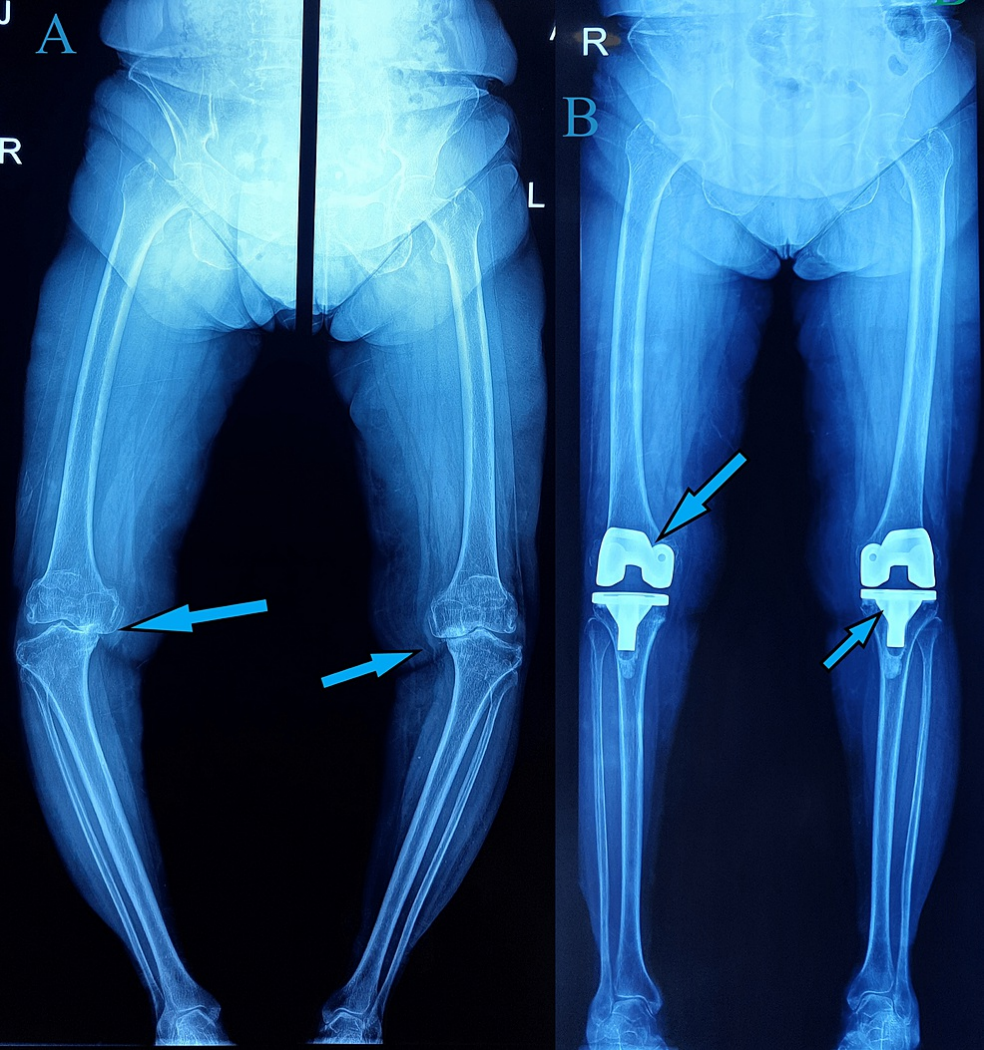
A) Preoperative hip-knee-ankle X-ray showing significant varus deformity and arthritis of both knee joints. B) Postoperative hip-knee-ankle X-ray showing corrected alignment and well-positioned implants

**Figure 9:**
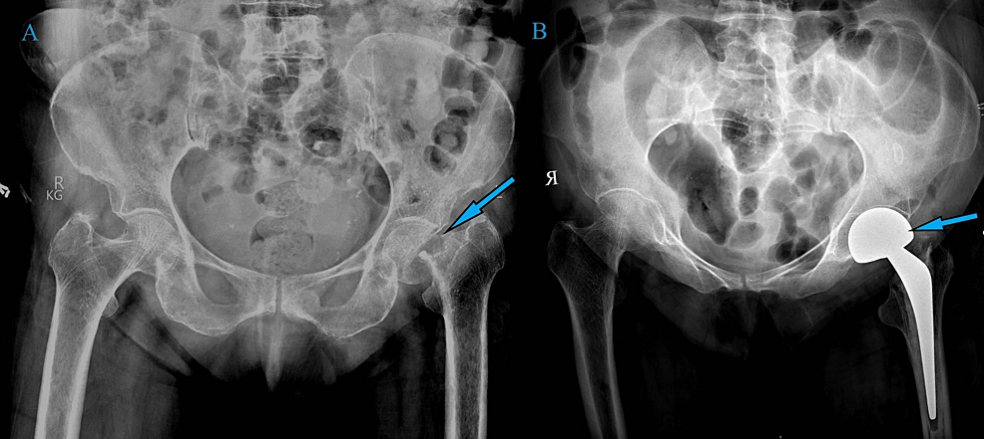
A) Preoperative pelvic X-ray showing displaced left femur neck fracture with significant osteoporosis B) Postoperative pelvic X-ray showing cemented left hip bipolar hemiarthroplasty at six-month follow-up

A 69-year-old female, 76kg in weight, 176cm in height, and 31.2kg/m2 BMI with bilateral knee rheumatoid arthritis underwent TKA using a cemented posterior stabilized prosthesis (PFC Sigma, Depuy Synthes, Warsaw, IN) (Figures 8A-8B). Preoperatively, she had a 25-degree deformity on both knees. She was on chronic low-dose steroids. Three months after TKA, she had no pain and was ambulating comfortably. Five months after TKA, she had increasing left groin pain while walking. She visited a nearby hospital, and her X-ray examination showed normal findings. A few weeks later, she suddenly felt severe pain in the left groin area while walking and fell to the ground. An X-ray of the hip showed a displaced fracture of the left femur (Figure 9A). DEXA of the hip and lumbar spine showed a T-score of -2.8 and -2.5. We managed operatively with cemented bipolar hemiarthroplasty (C-Stem, DePuy Synthes, Warsaw, IN) (Figure 9B). 

Case 4

**Figure 10:**
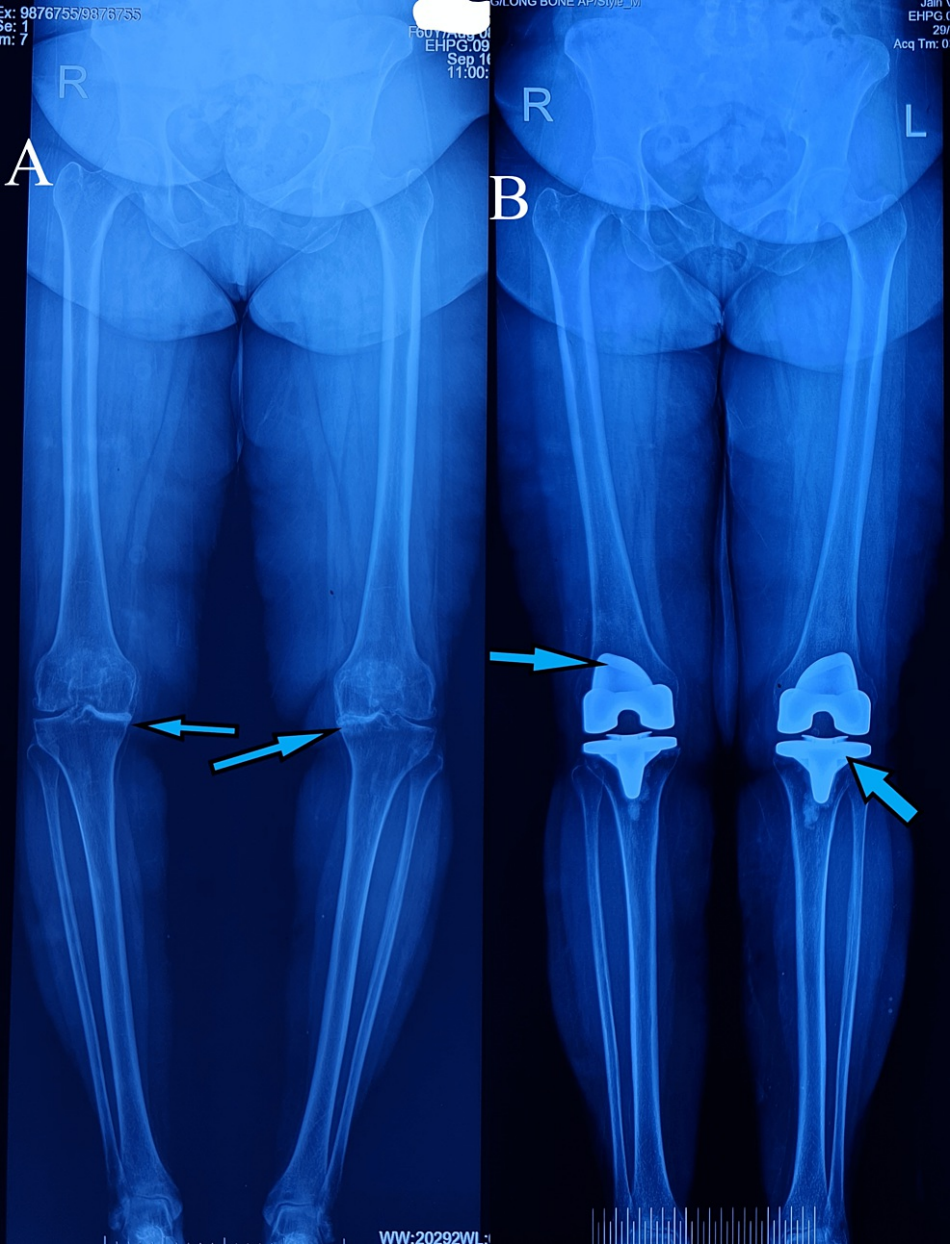
A) Preoperative hip-knee-ankle X-ray showing varus deformity and arthritic knee joints. B) Postoperative hip-knee-ankle X-ray showing corrected alignment and well-positioned implants at six-week follow-up

**Figure 11:**
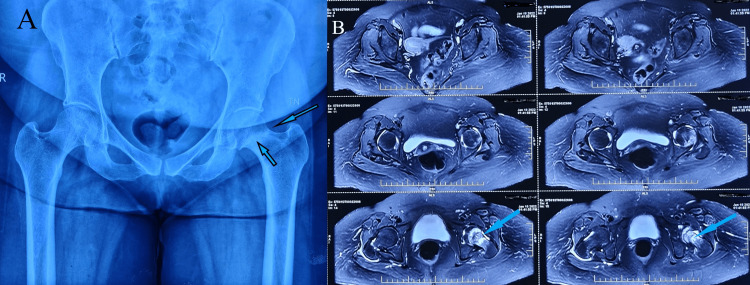
A) Pelvic X-ray showing sclerotic line suggestive of a stress fracture of the left femur neck. B) MRI of the pelvis showing complete stress femur neck fracture, bone edema, and intra-articular hip effusion

**Figure 12:**
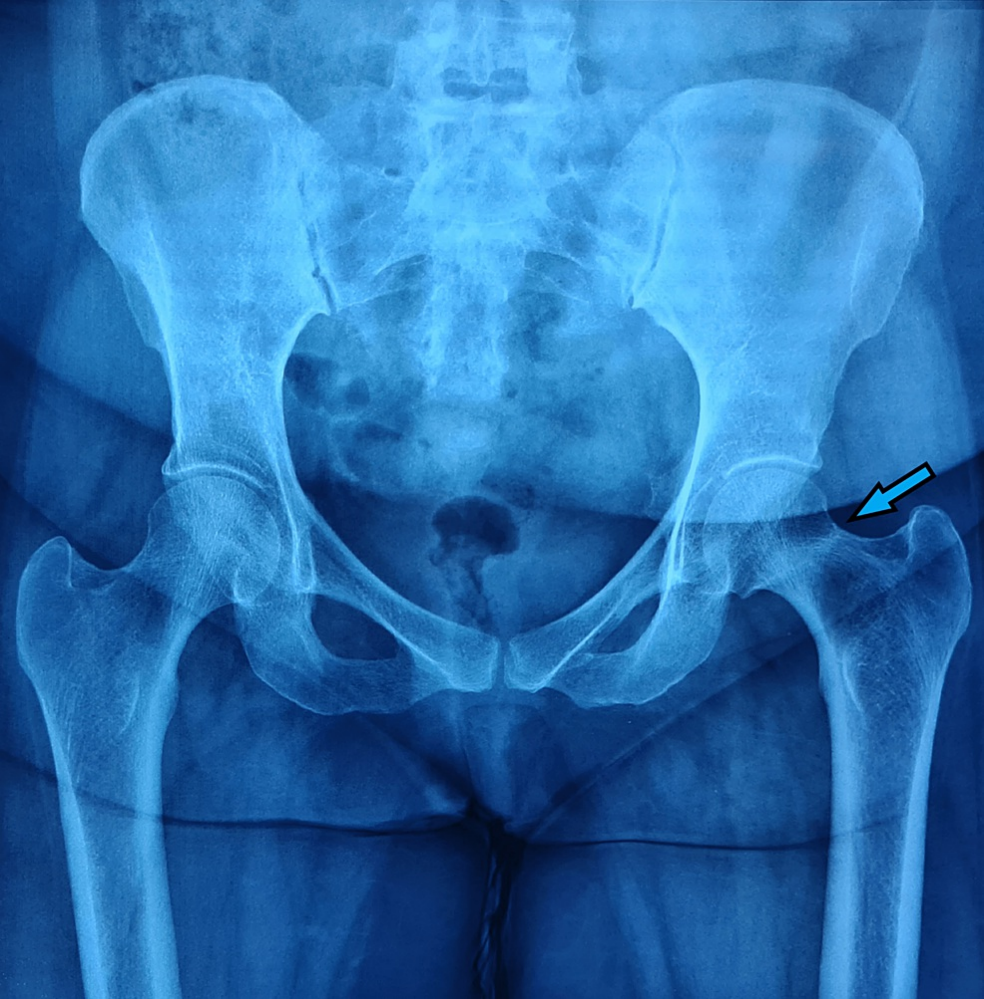
A six-month follow-up pelvic X-ray showing healed fracture of stress femur neck fracture after non-operative treatment

A 60-year-old female, 76kg in weight, 158cm in height, and 30.4kg/m2 BMI underwent surgery for bilateral knee osteoarthritis with TKA using a posteriorly stabilized prosthesis (Attune Depuy Synthes, Warsaw, IN) (Figures 10A-10B). The postoperative course was uneventful. The patient complained of left hip pain after five months of knee replacement. A pelvic X-ray revealed a sclerotic line, suggesting a non-displaced left femur stress fracture (Figure 11A). MRI showed a linear hypointense line running along the sub-capital region and intra-articular hip effusion (Figure 11B). A DEXA of the hip and lumbar spine showed a T-score of -2.9 and -1.4. Despite being advised to have a surgical fixation, she refused surgery and agreed to complete bed rest for two months, followed by gradual weight-bearing. At six months follow-up after the stress fracture of the femur neck, the patient was pain-free and showed radiologic signs of bone healing (Figure 12).

Case 5 

**Figure 13:**
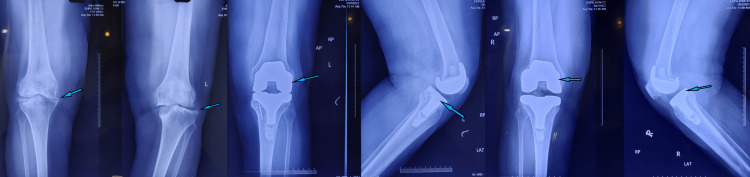
A) Preoperative X-ray showing varus deformity and arthritic knee joints B) Postoperative X-ray of the left knee showing good alignment and implant positioning C) Postoperative X-ray of the right knee showing good alignment and implant positioning

**Figure 14:**
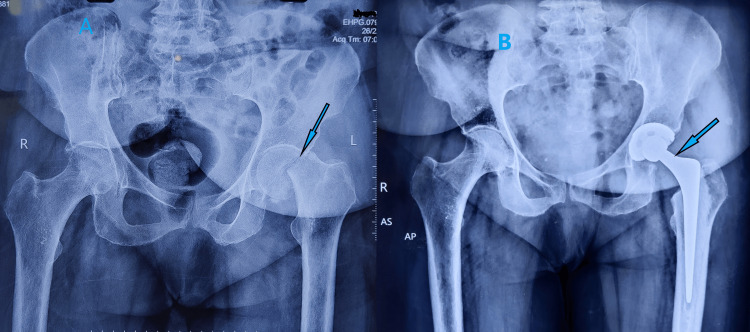
A) Preoperative pelvic X-ray showing displaced left subcapital femur neck fracture and B) Immediate postoperative pelvic X-ray showing well-positioned cemented total hip replacement implants

A 67-year-old female patient underwent surgery for bilateral knee rheumatoid arthritis with TKA using a posterior stabilized prosthesis (PFC Sigma, Depuy Synthes, Warsaw, IN) (Figures 13A-13C). She was comfortable with the postoperative course. The patient complained of left hip pain after five months of knee replacement. She visited a nearby hospital, and the initial pelvic X-ray was normal. A month after her hip pain began, she tripped and felt a sudden worsening pain that rendered her unable to walk. An X-ray revealed a displaced fracture of the femoral neck (Figure 14A). A DEXA of the hip and lumbar spine showed a T-score of -2.6 and -1.9. We managed operatively with a cemented total hip replacement (Figure 14B). 

For all cases, we administered teriparatide injection of 20mcg SC daily for one year for osteoporosis and supplemented them with calcium and vitamin D. 

## Discussion

Stress fracture of the femoral neck following TKA is rare, with only a few reported cases in the English literature [8,9]. Various authors have described associated factors related to stress fractures of the femur neck after TKA in the literature. Advanced age, female gender, high body mass index, the patient's increased level of activity following TKA, osteoporosis, rheumatoid arthritis, prolonged steroid use, preoperative valgus deformity of the knee, altered biomechanics after TKA, and implantation of a rotating hinge prosthesis are the associated risk factors for these fractures [2-6].

In our cases, the age ranged from 55 to 69 years, with an average age of 62.2 years. All of our patients are female. Three (cases 1, 3, and 5) of them had rheumatoid arthritis; all of them had osteoporosis; and four (cases 1, 2, 3, and 4) of them were obese. All cases had preoperative varus deformity of the knee, and case 3 had a significant knee deformity of 25 degrees. We used cemented, unconstrained implants in all patients for TKA. Three (cases 1, 3, and 5) of the cases had taken steroids for prolonged periods. We assume that the main predisposing factor for the fracture in our patients might be an increase in the level of activity in the osteoporotic bone after a period of relative immobility. Prolonged intake of steroids might have also contributed to the fractures in cases 1, 3, and 5. A significant correction of preoperative deformity in case 3 may also be a predisposing factor.

The literature describes the time at which the stress fracture occurred after TKA as ranging from 2-33 months, with an average of 8.7 months [9]. In our cases, the time ranges from 4-6 months after TKA, with an average of 4.9 months. Hence, we defined a stress fracture after TKA as a non-traumatic fracture that appears in the femoral neck within six months of the procedure. 

According to Kim KK et al., TKA significantly lowers proximal femur bone mineral density (BMD) in the first three months postoperative period because of less postoperative activity [10]. All of our cases had osteoporosis on DEXA scanning during the diagnosis of the stress fracture. We believe it might be wise to screen all patients undergoing TKA with DEXA and start osteoporosis treatment as early as possible after TKA to prevent such complications.

Bernatz JT et al. reported that the prevalence of unrecognized osteoporosis is significantly higher (36.7 percent in the study) with a well-functioning TKA [11]. From our experience, most of our patients come for TKA late in the advanced arthritis stage and after a long period of immobility with low BMD. We believe that the cause of osteoporosis in our cases may relate to the pre-and postoperative relative inactivity of the patient and the steroid intake (cases 1, 3, and 5).

Tatsuya S. et al. reported that teriparatide administration increases periprosthetic bone mineral density after TKA [12]. We used teriparatide in all our cases of osteoporosis, and all of them responded well.

Though radiographs are the initial workup to diagnose a stress fracture, 90% of initial X-rays may not reveal evidence of a stress fracture, requiring an MRI evaluation to rule out the diagnosis [7]. In our cases, cases 3 and 5 had hip pain and normal initial X-ray findings and developed the fracture later. The treating doctor should always consider a stress fracture and must not hesitate to request an MRI in a patient with gradual onset hip pain, no history of trauma, and normal pelvic radiographic findings.

Bernstein EM. et al. described that the updated management of stress femoral neck fractures depends on the findings of the MRI morphology of the fracture and intra-articular hip effusion [7]. Compression-sided fractures with less than 50 percent of the fracture line and no hip effusion are the indications for nonoperative treatment. Compression-sided fractures, over 50 percent fracture line, or intra-articular hip effusion are the indications for prophylactic operative fixation to prevent the progression of the fracture and displacement. A tension-sided fracture is an indication of operative fixation [7].

In our cases, we managed the first case surgically with cannulated hip screws as the fracture was on the tension side with a high risk of progression to displacement. Despite our advising surgical fixation in cases (2 and 4), they preferred nonoperative treatment and six-month follow-ups showed pain-free hip and radiological signs of healing. Cases 2 and 4 demonstrated that if an early diagnosis is made before the fracture is complete, it can still be managed conservatively if the patient desires so. As the presentation was a displacement of a neck fracture, we treated case 3 with a cemented bipolar hemiarthroplasty and case 5 with a cemented total hip replacement. The displacement of the fractures and subsequent joint replacement surgery in these cases might have been preventable if the primary treating doctor considered the stress femur neck fracture as a differential diagnosis. Early diagnosis and management may help in preventing further displacement of the fracture, long-term disability, and unnecessary hip replacement surgeries.

## Conclusions

A stress fracture of the femoral neck following TKA is rare. A high degree of suspicion and early diagnosis are the keys to avoiding displacement of the fracture and undesired hip replacement surgeries. Preoperative screening of bone mineral density with DEXA during TKA may help in the early diagnosis and initiation of osteoporosis treatment.
